# Supplementary material for: Microfluidic-based mini-metagenomics enables discovery of novel microbial lineages from complex environmental samples
Source: eLife. 2017 Jul 5;6:e26580. doi: 10.7554/eLife.26580 (PMC5498146; doi:10.7554/eLife.26580)
Supplement: Supplementary file 1. — DOI: http://dx.doi.org/10.7554/eLife.26580.024 [file elife-26580-supp1.docx]

Supplementary file 1: COG terms used for marker gene based phylogenetic tree

| **COG id** | **Gene name** |
| --- | --- |
| COG0013 | alanyl-tRNA_synthetase |
| COG0016 | phenylalanyl-tRNA_synthetase_alpha_subunit |
| COG0018 | arginyl-tRNA_synthetase |
| COG0048 | ribosomal_protein_S12 |
| COG0049 | ribosomal_protein_S7 |
| COG0051 | ribosomal_protein_S10 |
| COG0052 | ribosomal_protein_S2 |
| COG0060 | isoleucyl-tRNA_synthetase |
| COG0072 | phenylalanyl-tRNA_synthetase_beta_subunit |
| COG0080 | ribosomal_protein_L11 |
| COG0081 | ribosomal_protein_L1 |
| COG0085 | DNA-directed_RNA_polymerase_beta_subunit (RpoB) |
| COG0086 | DNA-directed_RNA_polymerase_beta_prime_subunit (RpoC) |
| COG0087 | ribosomal_protein_L3 |
| COG0088 | ribosomal_protein_L4 |
| COG0089 | ribosomal_protein_L23 |
| COG0090 | ribosomal_protein_L2 |
| COG0091 | ribosomal_protein_L22 |
| COG0092 | ribosomal_protein_S3 |
| COG0093 | ribosomal_protein_L14 |
| COG0094 | ribosomal_protein_L5 |
| COG0096 | ribosomal_protein_S8 |
| COG0097 | ribosomal_protein_L6P |
| COG0098 | ribosomal_protein_S5 |
| COG0099 | ribosomal_protein_S13 |
| COG0100 | ribosomal_protein_S11 |
| COG0102 | ribosomal_protein_L13 |
| COG0103 | ribosomal_protein_S9 |
| COG0127 | Xanthosine_triphosphate_pyrophosphatase |
| COG0130 | Pseudouridine_synthase |
| COG0164 | ribonuclease_HII |
| COG0172 | seryl-tRNA_synthetase |
| COG0184 | ribosomal_protein_S15P |
| COG0185 | ribosomal_protein_S19 |
| COG0186 | ribosomal_protein_S17 |
| COG0193 | peptidyl-tRNA_hydrolase |
| COG0197 | ribosomal_protein_L16 |
| COG0198 | ribosomal_protein_L24 |
| COG0200 | ribosomal_protein_L15 |
| COG0201 | preprotein_translocase_subunit_SecY |
| COG0202 | DNA-directed_RNA_polymerase_alpha_subunit (RpoA) |
| COG0216 | protein_chain_release_factor_A |
| COG0233 | ribosome_recycling_factor |
| COG0244 | ribosomal_protein_L10 |
| COG0255 | ribosomal_protein_L29 |
| COG0256 | ribosomal_protein_L18 |
| COG0343 | queuine/archaeosine_tRNA-ribosyltransferase |
| COG0481 | membrane_GTPase_LepA |
| COG0495 | leucyl-tRNA_synthetase |
| COG0504 | CTP_synthase |
| COG0519 | GMP_synthase_PP-ATPase_domain |
| COG0532 | translation_initiation_factor_2 |
| COG0533 | metal-dependent_proteases_with_possible_chaperone_activity |
| COG0541 | signal_recognition_particle_GTPase |
| COG0691 | tmRNA-binding_protein |
| COG0858 | ribosome-binding_factor_A |
